# Enhanced Persistency of Resting and Active Periods of Locomotor Activity in Schizophrenia

**DOI:** 10.1371/journal.pone.0043539

**Published:** 2012-08-28

**Authors:** Wataru Sano, Toru Nakamura, Kazuhiro Yoshiuchi, Tsuyoshi Kitajima, Akiko Tsuchiya, Yuichi Esaki, Yoshiharu Yamamoto, Nakao Iwata

**Affiliations:** 1 Department of Psychiatry, Fujita Health University School of Medicine, Aichi, Japan; 2 Educational Physiology Laboratory, Graduate School of Education, The University of Tokyo, Tokyo, Japan; 3 Department of Psychosomatic Medicine, Graduate School of Medicine, The University of Tokyo, Tokyo, Japan; Baylor College of Medicine, United States of America

## Abstract

Patients with schizophrenia frequently exhibit behavioral abnormalities associated with its pathological symptoms. Therefore, a quantitative evaluation of behavioral dynamics could contribute to objective diagnoses of schizophrenia. However, such an approach has not been fully established because of the absence of quantitative biobehavioral measures. Recently, we studied the dynamical properties of locomotor activity, specifically how resting and active periods are interwoven in daily life. We discovered universal statistical laws (“behavioral organization”) and their alterations in patients with major depressive disorder. In this study, we evaluated behavioral organization of schizophrenic patients (n = 19) and healthy subjects (n = 11) using locomotor activity data, acquired by actigraphy, to investigate whether the laws could provide objective and quantitative measures for a possible diagnosis and assessment of symptoms. Specifically, we evaluated the cumulative distributions of resting and active periods, defined as the periods with physical activity counts successively below and above a predefined threshold, respectively. Here we report alterations in the laws governing resting and active periods; resting periods obeyed a power-law cumulative distribution with significantly lower parameter values (power-law scaling exponents), whereas active periods followed a stretched exponential distribution with significantly lower parameter values (stretching exponents), in patients. Our findings indicate enhanced persistency of both lower and higher locomotor activity periods in patients with schizophrenia, probably reflecting schizophrenic pathophysiology.

## Introduction

Many psychiatric diseases, including depression and schizophrenia (SCZ), result in behavioral alterations [1]. Indeed, the diagnostic criteria for SCZ include symptoms related to behavioral abnormalities [1], [2], such as disorganized behavior, motor retardation, and catatonic behavior. In addition to these clinical criteria, various behavioral abnormalities have recently been reported, including psychomotor slowing [3] or neurological soft signs [4], [5], [6]. Therefore, the behavioral dynamics involving the above features is considered to be quite complex and contain rich information on pathological symptoms of SCZ.

Actigraphy has been widely used in clinical fields as an objective and noninvasive tool to measure spontaneous physical activity in daily life and has significant potential in diagnosing psychiatric disorders [7], [8]. Early pioneering work in this field demonstrated alterations in activity levels as well as chronobiological disturbances in patients suffering from depression [8], [9]. In SCZ patients, Berle et al. reported that the daily activity levels decrease, as seen in patients with depression, whereas behavioral patterns are more structured compared to patients with depression in terms of inter-daily stability and intra-daily variance of locomotor activity levels [10]. From the viewpoint of dynamical properties, Hauge et al. recently examined an entropy measure to show the increase in complexity of minute-by-minute activity fluctuations in SCZ patients [11]. Furthermore, the immobility duration in locomotor activity, possibly reflecting psychomotor slowing [3], successfully distinguishes subtypes of SCZ [12] is associated with motor retardation [13]. Hence, assessing locomotor activity may be useful in quantitatively evaluating psychiatric diseases associated with behavioral abnormalities. However, the above approaches have not yet been fully established because these activity measures are likely to be affected by various environmental, methodological, and lifestyle factors. In addition to the lack of robustness as a result of differences in study conditions, it is still difficult to directly connect the traditional measures to underlying pathophysiology, whereas several studies based on neuroimaging approaches have recently suggested the existence of an association with brain structures or functions [14], [15], [16], [17].

We recently studied the dynamical properties of locomotor activity in both humans and mice and discovered robust and identical statistical laws of behavioral organization, specifically how resting and active periods derived from locomotor activity are interwoven in daily life [18], [19]. Furthermore, we also found shared alterations in the resting period statistical law in humans with major depressive disorder (MDD) [18] and mice with an eliminated circadian clock gene (period 2) [19]. These findings suggest the presence and the robustness of an underlying principle governing behavioral organization across species and are expected to facilitate the understanding of the pathophysiology of neurobehavioral diseases. More importantly, the possibility of cross-species translation shown through a behavioral organization analysis may play a crucial role in bridging the gap between specific genetic substrates and behavioral endophenotypes in psychiatric disorders.

Therefore, in this study, we investigated the dynamical properties of locomotor activity in SCZ patients and show that their locomotor activity in daily life is well characterized by systematically longer durations of both lower and higher activity levels, indicating their enhanced persistency.

## Results

### Alterations in Locomotor Activity in Schizophrenia

**Figure 1:**
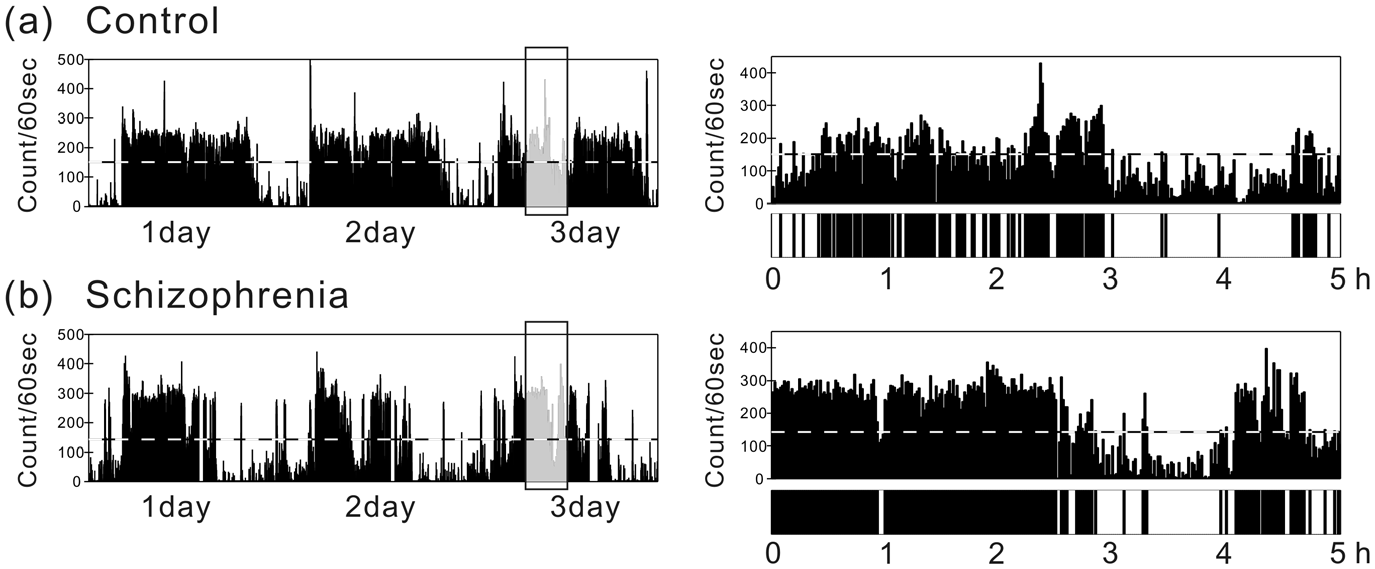
Fluctuation in locomotor activity. Illustrative examples of locomotor activity data for a control (a), and a schizophrenia (SCZ) patient (b) over three consecutive days (left panels). The right panels are magnifications of the left panels with 5-h periods during the third day. The overall average of non-zero activity counts was used as the threshold (horizontal dotted line), and the period during which the counts were successively below or above the threshold is coded as a resting (white bar in bottom panels) or active (black bar) period, respectively.

Figure 1 shows typical examples of locomotor activity data for a control and SCZ patient over three consecutive days. During daytime, locomotor activity of the control was characterized by consistently higher levels of activity, whereas the patient exhibited intermittent bursts in activity counts with more episodes of slowing down or cessation of movement (Fig. 1, left panels). Such a difference resulted in a significant increase (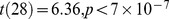) in standard deviation (SD) of locomotor activity for patients (Table 1). In contrast, higher locomotor activity tended to persist longer in patients compared with that in controls once it was initiated (Fig. 1, right panels). The duration of higher activity persistently lasting >1 h is presented in Fig. 1(b). These alterations in behavior patterns are quantitatively characterized by parameters of resting and active period distributions in the next section.

**Table 1:** Basic statistics and behavioral organization parameters of locomotor activity.

	Mean[count/min]	SD[count/min]	Resting period 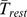 [min]	Active period 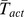 [min]	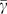	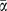	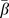
Control	130.9±6.1	**101.5±1.4**	**7.7±0.4**	6.6±0.3	**0.99±0.03**	0.39±0.02	**0.64±0.02**
Schizophrenia	121.2±5.2	**115.8±1.8^†^**	**11.6±0.7^†^**	7.5±0.6	0.86±0.03^*^	0.46±0.03	0.57±0.02^*^

Values are mean ± SEM. **^†^** and **^*^ indicate 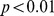 and 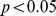 from controls, respectively. The results for controls are reproduced from our prior work [18], [19].**

### Cumulative Distributions of Resting and Active Periods

We estimated the cumulative distribution 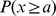 of the durations 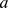 (min) of both resting periods, where the activity counts were successively lower than a certain predefined threshold value, and of active periods, where the counts were successively higher than the threshold value. In this study, we have described the results when an overall average of non-zero activity counts is used as the threshold value and then discuss the effects of threshold values.

**Figure 2:**
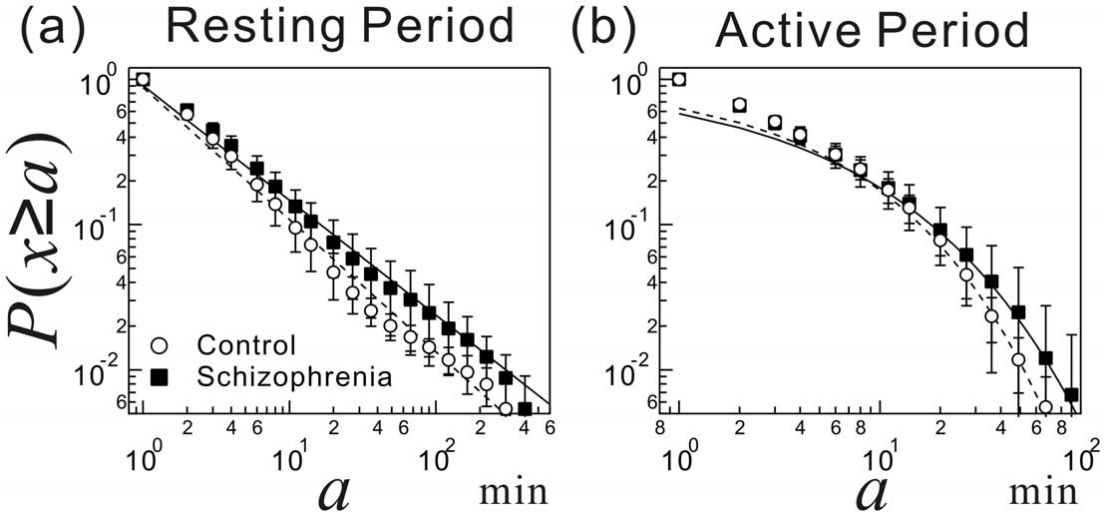
Alterations in behavioral organization parameters in schizophrenic patients. Cumulative distributions of resting and active period durations in locomotor activity for healthy subjects and schizophrenia (SCZ) patients. (a) Cumulative distributions 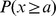 of the resting period durations 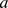 for control group (white circles) and SCZ group (black diamonds). Error bars indicate standard error of the mean. Straight lines are eye guides with the overall mean values; 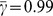 for healthy subjects and 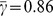 for the patients. (b) The same as (a) but for the active period durations. The solid curves are stretched exponential functions with the group mean parameter values in Table 2. Note that these distributions are plotted with wider bins (>1 min) for the purpose of illustration.

Figure 2 shows the average cumulative distributions of resting and active period durations for both groups. The distribution of resting periods for SCZ patients was higher compared with that for controls (Fig. 2a), particularly at longer durations, implying more frequent episodes of longer resting period in the patients. The resting period distributions for healthy subjects and SCZ patients took a power-law form,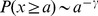, for over more than two decades (2 min–200 min) with significantly different scaling exponents of 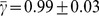 for controls and 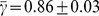 for SCZ patients (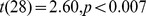). This was associated with a significantly longer mean resting period duration, 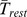, in the patients (11.6±0.7 min, 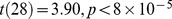) compared with that in controls (7.7±0.4 min), suggesting more episodes of slowing down or cessation of movement in daily life of SCZ patients (Table 1).

**Table 2:** Demographic and clinical characteristics of the schizophrenic patients.

	Schizophrenia
Age (yrs)	38.5±8.4
Gender (Male/Female)	9/10
Chlorpromazine equivalents (mg)	509.5±372.8
PANSS	
Total score	72.0±16.6
Positive scale	15.8±5.5
Negative scale	18.9±5.3
General Psychopathology scale	37.3±9.1
Five-factor model	
Positive symptoms	15.2±5.1
Negative symptoms	19.2±6.2
Disorganization symptoms	25.0±6.6
Excitement	16.2±4.0
Emotional distress	41.1±11.4
DIEPSS	2.1±1.8

PANSS; Positive and Negative Syndrome Scale, DIEPSS; Drug-Induced Extrapyramidal Symptoms Scale.

Values are mean ± S.D.

Although no significant difference in mean active period duration, 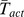, was observed between the groups (Table 2), the average cumulative distribution of the active period durations for SCZ patients had a fatter tail in the longer duration range, particularly in the range of >20 min compared with the average cumulative distribution of the active periods for controls (Fig. 2b). This finding indicates that patients tend to continuously maintain their activity at higher levels once it is initiated. The cumulative distributions of the active periods for both groups were well approximated by a stretched exponential functional form, 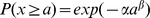, for a wide range of time scales with significantly different stretching parameters of 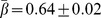 for controls and 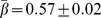 for SCZ patients (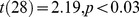), which is suggestive of enhanced persistence of higher activity levels in the patients (Table 1).

### Dependency of the Distribution Parameters on Threshold Values

**Figure 3:**
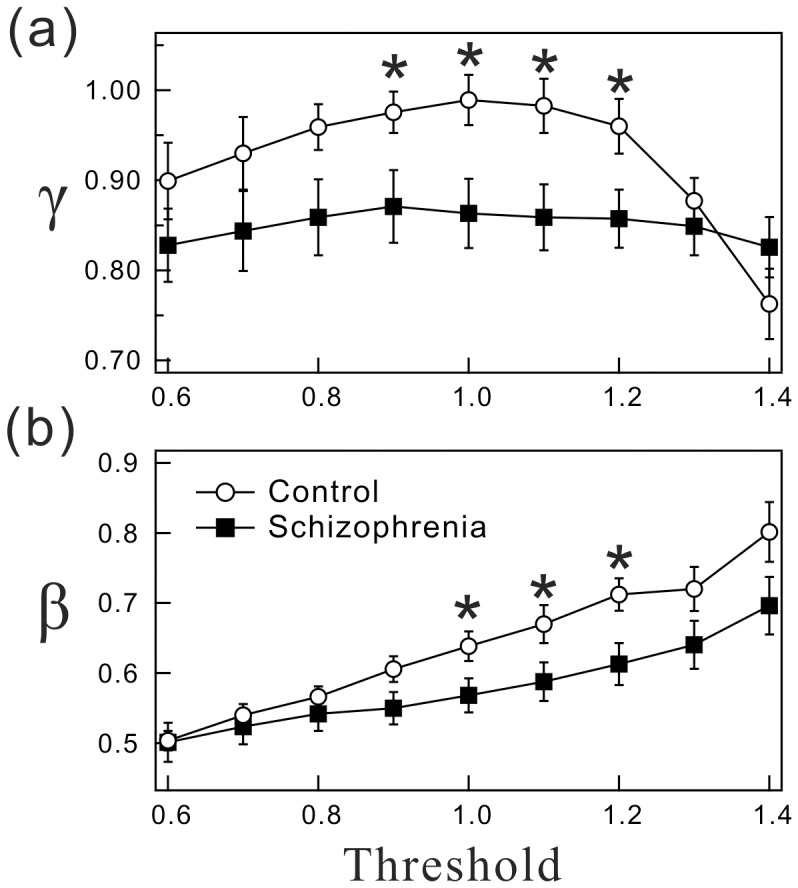
Dependency of distribution parameters on threshold values. (a) The values of 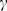 with different threshold values of 0.6, 0.7, 0.8, 0.9, 1.0, 1.1, 1.2, 1.3, and 1.4 times the overall average for non-zero activity counts. (b) The same as (a) but for 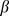. Bars indicate standard error of the mean and the asterisks indicate statistically significant differences between the groups.

By definition, the choice of threshold values affects resting and active period durations (the higher the threshold, the longer the mean resting period and the shorter the active period) and thus their distributions. Therefore, we evaluated the effects of threshold values on the results. Figure 3 shows the values of the distribution parameters 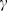 and 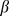 with different threshold values ranging from 0.6 to 1.4 times the overall mean of non-zero activity counts. The cumulative distributions of resting and active periods were respectively well fitted by a power-law form and a stretched exponential functional form for all threshold values examined. The resulting estimate 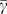 of the resting period distributions in the patients was significantly smaller compared with that in controls over the range of 0.9–1.2 (Fig. 3a), and the value of 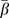 for the active period distributions was consistently smaller over the range of 1.0–1.2 (Fig. 3b). These findings indicate that the results based on the distribution parameters are largely unaffected by the choice of threshold values.

### Association with Clinical Scores

We conducted a stepwise regression analysis to examine the relationship between the measures of locomotor activity listed in Table 1 and clinical scor\es on the Positive and Negative Syndrome Scale (PANSS) (total, positive scale, negative scale, general psychopathology scale), five-factor model (positive symptoms, negative symptoms, disorganization, excitement, emotional distress), and the Drug-Induced Extrapyramidal Symptoms Scale (DIEPSS). The regression analysis demonstrated that only mean activity counts during the study period were significantly and positively correlated with negative symptom scores on PANSS (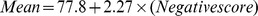, 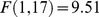, 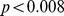, 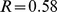), whereas the other locomotor activity measures had no significant correlation with the clinical scores after controlling for age. We also examined the gender effects on locomotor measures but did not find any significant relationship with clinical scores.

Furthermore, we considered the effects of medications on behavioral dynamics. The chlorpromazine-equivalent dosage was significantly and positively correlated with the stretching parameter 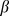 (Pearson’s correlation coefficient: 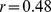, 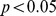) and increasing tendency with age (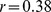, 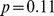). Because 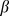 was also significantly correlated with age (see Methods), we further tested the possibility of a spurious relationship between 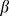 and dosage. The partial correlation without the age effect was not significant (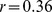, 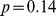), suggesting no direct association between them. In the stepwise regression for 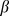, after controlling for age, dosage was not found to be a significant parameter.

## Discussion

### Behavioral Abnormalities in Schizophrenia

We found that SCZ patients, similar to MDD patients [18], demonstrated systematic and significant increase in resting period over a wide range of durations, indicating frequent episodes of slowing down or cessation of movement which in turn indicates psychomotor slowing in schizophrenia [3]. Different from MDD, active period in SCZ patients exhibited enhanced persistency, indicating that such patients have the tendency to continue their activity once it is started, possibly reflecting excessive motor activity, repetitive movements, and psychomotor agitation [1]. Notably, this is the first demonstration of the alteration in active-period behavioral organization in patients with a psychiatric disorder, whereas we reported its universality in healthy and depressed humans [18] as well as in wild-type and circadian clock gene knockout mice in our previous study [19]. Although the specific clinical symptoms of alterations in behavioral laws in resting and active periods are still unclear, the changes in locomotor dynamics in SCZ patients may have originated from the integration and/or combination of the above mentioned behavioral abnormalities.

### Correlation with Clinical and Demographic Variables

Our interpretations should be considered with caution because of the lack of significant correlations with clinical scores. The only significant correlation we found was a positive correlation between mean activity levels and PANSS negative scores. However, this was rather counterintuitive and contradictory to the results of previous studies [12], [13], [20]. The inconsistency of this finding may come from the effects of inclusion of sleep time and the small sample size as well as heterogeneity of the patients. Alternatively, considering that many studies failed to find significant relationships between actigraphic parameters and clinical scores [21], measures of behavioral dynamics do not have direct and/or consistent relationships with SCZ symptoms, and the measures may not be a state marker but a trait marker.

The aging effect on the stretched parameter 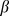 of the active period distributions should be addressed. Our results showed that the value of 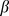 in SCZ patients increased with age, indicating less of a difference in persistency from controls. In other words, this tendency implies that the abnormal persistency of active periods gradually normalizes with age. In our experience, schizophrenic symptoms basically do not show improvement with age, whereas psychosocial cognition exhibits a significant improvement. Indeed, prior clinical studies have reported significant improvement in personal system variables, including psychosocial ability related to the functioning level in older adults with SCZ [22], [23], [24], [25]. Although specific aging effects on neurological and biological systems remain unknown, it is thought that aging plays an important role improving the illness and consequently causing alterations in behavioral dynamics. The aging effect should be investigated when considering our behavioral measures as a trait marker, and this issue should be addressed in future research.

### Limitations of Our Study

A potential limitation of the our study is the effects of antipsychotic medications on behavioral dynamics [20], [26], [27], [28]. Because the efficacy of antipsychotics is thought to be mediated mainly by blocking D_2_ receptors in the striatum of the basal ganglia, which plays an important role in motor control [29], [30], antipsychotic medications may influence activity measures through the striatum. For example, administration of olanzapine to healthy subjects leads to a decrease in overall mean activity levels due to an increase in immobility duration, even at a dose too low to cause clinically observable extrapyramidal side effects [26]. Another atypical antipsychotic drug, risperidone, also decreases activity levels in SCZ patients as compared to healthy subjects, although there is no significant difference in the effect with olanzapine among patients [20]. In contrast, Walther et al. demonstrated that SCZ patients treated with olanzapine have higher activity levels as compared to those treated with risperidone [28]. From a viewpoint of locomotor rhythm patterns, administration of clozapine results in rigidly entrained rest-activity cycles with distinctive onset and offset of activity, whereas classical neuroleptics (haloperidol or flupentixol) cause minor to major circadian rhythm abnormalities [27]. Therefore, we cannot exclude the possibility that the alteration in behavioral organization originates from the effects of medications on behavioral dynamics.

However, after controlling for age, we did not find a correlation between chlorpromazine-equivalent dosage and any of the activity measures. In addition, our previous study on MDD did not show any alteration in active period distributions or an association with the drug [18]. Therefore, our findings cannot be confirmed simply on the basis of the effects of the drugs. Thus, future studies on drug-free patients are required to address this issue. Another weakness of our study is the small sample size. Thus, it is necessary to conduct a large population study to generalize our findings.

### Effects of Sleep

It might be important to address the effects of sleep parameters, such as sleep duration or existence of daytime naps, on resting period distributions. Firstly, the common sleep events detected by continuous immobility and/or low activity counts in locomotor activity data may usually last for more than one hour. Even if such sleep events are fragmented as often observed in SCZ patients [31], almost all sleep durations would be more than 20–30 min, and thus the number of events disrupted within 20 min should be limited. Therefore, the range of time scales where the sleep parameters have significant effects on the shape of resting period distributions should be at longer durations, and those effects could be statistically minimal at smaller durations if there are. In addition, mathematically speaking, if such the effects are considerably dominant, the distributions might be distorted from a power law form, and possibly follow bi- or multimodal distributions with significant peaks in longer time scales. However, our findings were not the case: the clear power-law distributions which started from approximately 2 min and systematically lasted until approximately 200 min in both groups. Thus, we consider such the effects are limited in the range of durations we focus on.

Alternatively, it might be possible to consider removing such sleep events from the analysis based on some empirical filtering methods, such as Cole–Kripke algorithm [32]. This empirical method provides the period of time during which the mean of weighted neighboring activity data is less than a certain threshold value, however, it is not necessary to ensure that the estimated period is “true sleep”. Actually, this approach misjudges a certain state with psychomotor retardation as “sleep”, although such a symptom is extremely important clinically. Therefore, we consider that removing the actigraph-based sleep period could lead to loss of important information contained in locomotor activity, and thus, it is better not to adopt such an approach if patients do not have considerable sleep disorders, as is the case with our patients. Taking into consideration those facts, we used the entire recording period of locomotor activity in this study.

### Strengths of Our Approach

The major strengths of our approach are in the continuous and objective measurement of daily life activities and a quantitative evaluation of behavioral dynamics. The importance of prospective and longitudinal studies has been proposed because they provide novel insights into the pathophysiological processes of SCZ. In fact, several studies have examined the longitudinal developmental changes in potential endophenotypes or biomarkers mainly using neuroimaging techniques to capture pre-psychosis onset and the transition from the at-risk state to the illness or psychotic state [33], [34], [35], [36]. However, to accomplish these purposes, less costly and more easily implemented techniques are desirable if the methods are sensitive enough and can quantify performance sufficiently to detect onset and transition. Considering the merits of our method, such as objective/continuous recoding in a noninvasive way and the robust measures, our approach using actigraphy may be appropriate to conduct such longitudinal studies.

### Toward an Animal Model Based on the Endophenotype of Behavioral Dynamics

Developing animal models of human diseases is crucial for deeper understanding because the use of animal models allows researchers to study pathophysiology, symptomatic processes, effective drug development, novel therapies, and genetic substrates. One promising strategy to build animal models of mental illness is translating human behavior into animals [37], [38]. However, the translation of human behavioral abnormalities is quite difficult because of the absence of an established method to evaluate humans quantitatively. This critical drawback prohibits the systematic development of reliable animal models for psychiatric disorders; therefore, a practical solution is warranted.

We claimed recently that the statistical laws of behavioral organization provide quantitative and systematic measures to evaluate behavioral abnormalities across different species. Furthermore, we succeeded in identifying a mutant mouse with the circadian clock gene (period 2) that shares behavioral alterations with humans suffering from MDD [19]. We believe that this approach could provide a breakthrough for the above drawback and allow us to systematically explore animal models of SCZ. In particular, exploring animals that exhibit enhanced persistency of both resting and active locomotor activity periods could give rise to a partial model of the behavioral pathophysiology of SCZ patients. This approach may facilitate identification of genetic and/or neurological factors of the pathophysiology of SCZ as well as trajectories to SCZ. Furthermore, it could also be useful in revealing the effects of interactions with environmental factors and vulnerability to them.

### Conclusion

Behavioral abnormalities in SCZ patients can be well described by the enhanced persistency of both lower and higher activities, which is quite different from that in controls and MDD patients. We conclude that the behavioral organization of locomotor activity has potential as an objective biobehavioral measure for psychiatric diseases and that our findings could provide further insight into the pathophysiology of SCZ and contribute in the development of animal models.

## Materials and Methods

### Subjects

Nineteen SCZ patients (nine males, 10 females; 38.5 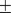 8.4 years of age) and 11 age- and gender-matched healthy subjects (five males, six females; 36.4±12.7 years of age) participated in this study. Demographic and clinical variables are given in Table S1 and Table S2. All SCZ patients were outpatients of Fujita Health University and met the Diagnostic and Statistical Manual of Mental Disorders (DSM-IV) criteria for SCZ [1] as confirmed by the Mini International Neuropsychiatric Interview. All healthy subjects were hospital employees at the University of Tokyo and were free of psychiatric disorders and medication.

### Inclusion and Exclusion Criteria

The inclusion criteria for SCZ were as follows: age of 20–65 years; SCZ as primary diagnosis; no physical comorbidity with significant effects on behavior. The exclusion criteria were as follows: any psychotic episode due to seizure or organic diseases, any DSM-IV Axis I disorders except SCZ, recent alteration in medication 2 weeks before the initiation of the study and during measurement of locomotor activity data, a score of ≥2 on any of the DIEPSS subscales, and high risk of suicide. All patients received antipsychotic medications (chlorpromazine-equivalent antipsychotics, mean dosage of 509 mg); however, the dosage and type of medication remained unchanged during the study period. None of the patients suffered from any chronic physical disorder or sleep disorder (see Text S1).

The patients were interviewed by trained psychologists and assessed using PANSS and DIEPSS before recording of the daily activity data. A five-factor model for the PANSS items [39] was also evaluated in this study (Table 2).

### Ethical Statement

Written informed consent was obtained from all subjects after they were fully informed about the purpose, procedures including actigraphy recording, and risks/benefits of the study. As for SCZ patients, psychiatrists who were in charge of the patients confirmed their capacity to consent; any other person, such as caregiver or next of kin, was not needed to consent on the behalf of the patients. This study was approved by the ethics committee of Fujita Health University, and the University of Tokyo conformed to the principles outlined in the Declaration of Helsinki.

### Assessment of Locomotor Activity Data

The locomotor activity data, defined as counts of events in which an acceleration signal crosses a zero level within a predefined time, were acquired from SCZ patients and healthy subjects. All participants wore the Actigraph Mini-Motionlogger (Ambulatory Monitors Inc., Ardsley, NY, USA) [8] on the wrist of their nondominant hand for more than 7 days, providing sufficient data points for the robust estimates of locomotor measures [healthy subjects: 7.1±0.2 (6.9–7.7) days, SCZ patients: 14.5±5.3 (7.0–21.0) days]. This activity monitoring device is widely used in clinical fields and has the capability of detecting small changes in wrist acceleration (up to 0.01 G/rad/s) such that even slight movements by the subjects are registered. Counts crossing zero were accumulated every 1 min. The participants were instructed to wear the activity monitor at all times, except while bathing or during rigorous exercise. The activity data during the period when the device was removed were identified and excluded from analysis. The averaged number of such events was less than twice per day for both groups (healthy subjects: 1.0/day, SCZ patients: 1.4/day), and the ratio of removed data points was <4.5% of the total length for healthy subjects and <6.6% for patients without a significant difference (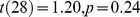). Note that the locomotor activity data of healthy subjects are essentially the same as those used in our previous studies [18], [19].

### Analysis of Locomotor Activity Data

To quantitatively evaluate locomotor activity patterns, we estimated the cumulative probability distribution 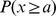 of durations 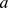 of both resting periods, where the activity counts were successively lower than a certain predefined threshold value, and of active periods, where the counts were successively higher than the threshold values [18], [19]. The cumulative probability distributions were obtained by numerically integrating the estimated probability density function 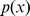 estimated from whole recording period with a bin width of 1 min as follows: 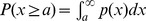.

Following our previous studies [18], [19], we assumed that cumulative distribution of active periods would assume a stretched exponential functional form 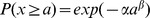 and that of resting period would assume a power law form 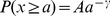. We fitted a stretched exponential function to the active period distributions based on the minimum of the chi-square statistic 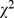 to obtain estimates for the fitting parameters 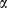 and 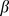. To estimate the scaling exponent 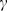 of resting distributions, we used the maximum likelihood method, which was recently proposed as an efficient method to provide accurate estimates of scaling exponents from empirical data following the power-law distribution [40]. Because this method provides a scaling exponent 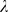 of the probability density function, 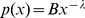 (not 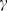 of the cumulative distribution 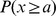), we subtracted 1 from the estimate 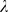 to obtain 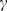 (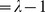). Based on our prior work [18], [19], we set the fitting range to 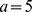 to 100 min for cumulative distributions of active periods and 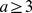 min for resting periods. Note that, by our numerical tests, one week continuous measurement is sufficient to robustly estimate the distribution parameters in these fitting ranges.

Although the choice of a power-law form for resting period distributions on the basis of high linearity in the log–log plots (see Fig. 2) is straightforward, there can be multiple choices of the model for active period distributions. Indeed, in our previous study [19], we fit other types of distributions; e.g., a power-law with exponential cut-off distribution 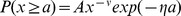 in addition to the stretched exponential form to active periods and then confirmed that the alternative models also fit the active period distributions quite well. Though the choice of the alternative model also provided a significant group difference in the parameter mainly determining the tail of the distribution (e.g., 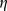 of a power-law with exponential cutoff distribution), we used the stretched exponential form for active period distributions in the present study to compare and discuss our results within the framework of our previous studies.

### Statistical Analysis

The Student’s *t*-test was used to compare the mean parameter values listed in Table 1. A repeated measures analysis of variance with Tukey’s post-hoc tests was performed to verify the effects of threshold values. The significance levels were corrected using Bonferroni adjustment for multiple comparisons. Furthermore, a multiple linear stepwise regression analysis was performed to examine the relationship between patients’ clinical characteristics and the locomotor activity measures. We considered scores on PANSS (total, positive symptom, negative symptom), five-factor model (positive symptom, negative symptom, disorganization, excitement, emotional distress), DIEPSS, and general psychopathology scale as independent variables. Because the stretching parameter 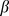 of active periods exhibited a significant correlation with subject age (Pearson’s correlation coefficient 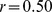, 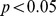), we also included it as an independent variable when constructing the model; 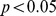 was considered significant. Note that for one subject, the scores of the five-factor model were not available because of loss of PANSS subscale items; therefore, we excluded this subject from the regression analysis.

## Supporting Information

Table S1 Demographics and medication of schizophrenia patients.
(DOC)

Table S2 Demographics of healthy subjects.
(DOC)

Text S1 Demographics and medication.
(DOC)
