# Supplementary material for: Enhanced Persistency of Resting and Active Periods of Locomotor Activity in Schizophrenia
Source: PLoS One. 2012 Aug 28;7(8):e43539. doi: 10.1371/journal.pone.0043539 (PMC3429496; doi:10.1371/journal.pone.0043539)
Supplement: Table S1 — Demographics and medication of schizophrenia patients. (DOC) [file pone.0043539.s001.doc]

**Table S1: Demographics and medication of schizophrenia patients.**

| **ID** | **Gender** | **Age**  **[yrs]** | **Duration of**  **illness [yrs]** | **PANSS**  **Total** | **PANSS**  **Positive** | **PANSS**  **Negative** | **PANSS**  **GP**^(a)^ | **DIEPSS** | **Medication** | **Anti**  **depressant** | **BZD** | **Occupation** |
| --- | --- | --- | --- | --- | --- | --- | --- | --- | --- | --- | --- | --- |
| **1** | M | 54 | 16 | 49 | 8 | 13 | 28 | 2 | CP 150mg, QTP 600mg | + | + | Unemployed |
| **2** | F | 40 | 16 | 81 | 22 | 25 | 34 | 6 | OLZ 15mg, CP50mg, BNS 16mg | + | + | Unemployed |
| **3** | F | 39 | 18 | 65 | 17 | 14 | 34 | 0 | SUL 150mg | + | + | Employed (part-time job) |
| **4** | M | 39 | 12 | 94 | 24 | 22 | 48 | 3 | TMP 12mg, OLZ 20mg | - | - | Unemployed |
| **5** | F | 54 | 21 | 67 | 12 | 20 | 35 | 4 | RIS 1.5mg | - | - | Unemployed |
| **6** | F | 42 | 12 | 66 | 10 | 20 | 36 | 0 | RIS 2mg | - | - | Unemployed |
| **7** | F | 26 | 9 | 74 | 22 | 18 | 34 | 0 | ARP 24mg | - | + | Unemployed |
| **8** | F | 35 | 15 | 69 | 21 | 15 | 33 | 3 | OLZ 20mg | - | - | Unemployed |
| **9** | F | 39 | 3 | 52 | 12 | 12 | 28 | 3 | OLZ 2.5mg | - | - | Unemployed |
| **10** | F | 37 | 16 | 56 | 12 | 13 | 31 | 4 | CZP 300mg | - | - | Unemployed |
| **11** | F | 22 | 5 | 35 | 8 | 8 | 19 | 2 | ARP 4mg | - | - | Student |
| **12** | M | 41 | 18 | 72 | 15 | 20 | 37 | 2 | RIS 2mg, ZTP 125mg | - | + | Unemployed |
| **13** | M | 25 | 3 | 76 | 17 | 19 | 40 | 0 | RIS 2mg | + | - | Unemployed |
| **14** | M | 47 | 20 | 78 | 17 | 20 | 41 | 4 | RIS 3mg | - | - | Unemployed |
| **15** | M | 39 | 19 | 83 | 23 | 23 | 37 | 3 | RIS 4mg | - | + | Unemployed |
| **16** | F | 42 | 3 | 98 | 24 | 21 | 53 | 0 | QTP 200mg | - | - | Unemployed |
| **17** | M | 32 | 14 | 101 | 13 | 29 | 59 | 4 | ARP 6mg | + | + | Unemployed |
| **18** | M | 38 | 6 | 79 | 11 | 25 | 43 | 0 | RIS 4mg | - | - | Unemployed |
| **19** | M | 41 | 5 | 72 | 12 | 22 | 38 | 0 | ARP 12mg | - | - | Employed (office worker) |

M, male; F, female; ARP, Aripiprazole; BNS, Blonanserin; CP, Chlorpromazine; CZP, Clozapine; OLZ, Olanzapine; PSP, Perospirone; QTP, Quetiapine; RIS, Risperidone; SUL, Sulpiride; TMP, Timiperone; ZTP, Zotepine; BZD, Benzodiazepine; ^(a)^ General Psychopathology
