# Supplementary material for: Enhanced Persistency of Resting and Active Periods of Locomotor Activity in Schizophrenia
Source: PLoS One. 2012 Aug 28;7(8):e43539. doi: 10.1371/journal.pone.0043539 (PMC3429496; doi:10.1371/journal.pone.0043539)
Supplement: Table S2 — Demographics of healthy subjects. (DOC) [file pone.0043539.s002.doc]

**Table S2: Demographics of healthy subjects.**

| **ID** | **Gender** | **Age**  **[yrs]** | **Occupation** |
| --- | --- | --- | --- |
| **1** | M | 30 | Employed |
| **2** | F | 28 | Employed |
| **3** | F | 32 | Employed |
| **4** | F | 60 | Employed |
| **5** | M | 30 | Employed |
| **6** | M | 25 | Employed |
| **7** | F | 34 | Employed |
| **8** | M | 23 | Employed |
| **9** | M | 35 | Employed |
| **10** | F | 44 | Employed |
| **11** | F | 59 | Employed |

M, male; F, female.
