# Supplementary material for: Enhanced Persistency of Resting and Active Periods of Locomotor Activity in Schizophrenia
Source: PLoS One. 2012 Aug 28;7(8):e43539. doi: 10.1371/journal.pone.0043539 (PMC3429496; doi:10.1371/journal.pone.0043539)
Supplement: Text S1 — Demographics and medication. (DOC) [file pone.0043539.s003.doc]

**Text S1: Demographics and medication**

1. **Patients with schizophrenia**

We have summarized the patient demographics and medication information in Table S1. None of the patients suffered from any chronic physical disorder or sleep disorder or had any distinguished motor side effect induced by medical admission (none of the DIEPSS items were >1). Their clinical picture was stable, and they had not changed the treatment for at least a few weeks before the study. They were categorized as having residual SCZ during the study period, although they had been initially diagnosed with paranoid or disorganized type on the basis of their clinical picture when they were admitted to the clinic for the first time. Note that we could not find any significant relationship between locomotor measures and the initial type diagnosed.

Of the 19 patients, 13 were unemployed and one was a university student. However, patients who retreated into their homes during the day or withdrew socially were not included in this study. Indeed, most of the unemployed patients received psychiatric daycare treatment as a rehabilitation program. In addition, 12 married women were able to perform routine housework by themselves. Therefore, they could perform almost all normal activities of daily living, and their activity levels were not considerably lower than those of healthy subjects. This is partly supported by the lack of significant difference in mean activity levels between groups (Table 1).

All patients took antipsychotics: typical antipsychotics (n = 4) and atypical antipsychotics [n = 15; risperidone (7/15), others (8/15)]. The patients treated with typical antipsychotics also received one or two atypical antipsychotics. Combination therapy with antidepressants was applied to seven patients. Benzodiazepines were administered to five patients as a sleep aid. But no considerable effect of the antidepressants or the benzodiazepines was observed on locomotor activity patterns.

1. **Healthy subjects**

The demographics for the healthy subjects are provided in Table S2. None of the healthy subjects suffered from any psychiatric disorder. These healthy subjects were age- and gender-matched controls (age: *p* = 0.58, *t*-test; gender: *p* = 0.61, Fisher’s exact test) with SCZ patients. All subjects were hospital employees at the University of Tokyo and were not familiar with the research hypotheses or the study design. Note that the locomotor activity data of healthy subjects were essentially the same as those used in our prior work [1,2].

1. Nakamura T, Kiyono K, Yoshiuchi K, Nakahara R, Struzik ZR, et al. (2007) Universal scaling law in human behavioral organization. Phys Rev Lett 99: 138103.

2. Nakamura T, Takumi T, Takano A, Aoyagi N, Yoshiuchi K, et al. (2008) Of mice and men--universality and breakdown of behavioral organization. PLoS One 3: e2050.
